# Facial Expressions Depicting Compassionate and Critical Emotions: The Development and Validation of a New Emotional Face Stimulus Set

**DOI:** 10.1371/journal.pone.0088783

**Published:** 2014-02-19

**Authors:** Kirsten McEwan, Paul Gilbert, Stephane Dandeneau, Sigrid Lipka, Frances Maratos, Kevin B. Paterson, Mark Baldwin

**Affiliations:** 1 School of Medicine, University of Cardiff, Cardiff, United Kingdom; 2 Mental Health Research Unit, Kingsway Hospital, Derby, United Kingdom; 3 Department of Psychology, Université du Québec à Montréal, Montreal, Quebec, Canada; 4 Centre for Psychological Research, University of Derby, Derby, United Kingdom; 5 College of Medicine, Biological Sciences and Psychology, University of Leicester, Leicester, United Kingdom; 6 Department of Psychology, McGill University, Montreal, Canada; University Hospital of Bellvitge-IDIBELL; CIBER Fisiopatología Obesidad y Nutrición (CIBERObn), Instituto Salud Carlos III; Department of Clinical Sciences, School of Medicine, University of Barcelona, Spain

## Abstract

Attachment with altruistic others requires the ability to appropriately process affiliative and kind facial cues. Yet there is no stimulus set available to investigate such processes. Here, we developed a stimulus set depicting compassionate and critical facial expressions, and validated its effectiveness using well-established visual-probe methodology. In Study 1, 62 participants rated photographs of actors displaying compassionate/kind and critical faces on strength of emotion type. This produced a new stimulus set based on N = 31 actors, whose facial expressions were reliably distinguished as compassionate, critical and neutral. In Study 2, 70 participants completed a visual-probe task measuring attentional orientation to critical and compassionate/kind faces. This revealed that participants lower in self-criticism demonstrated enhanced attention to compassionate/kind faces whereas those higher in self-criticism showed no bias. To sum, the new stimulus set produced interpretable findings using visual-probe methodology and is the first to include higher order, complex positive affect displays.

## Introduction

The evolution of attachment, affiliative and altruistic behaviour plays a central role in mammalian and human development [1]. With this has come the ability to recognise, process and respond to cues of altruistic, kind, and caring intentions and behaviours from others [2]. When it comes to friendships and long-term sexual relationships, humans are attuned to looking for altruistic individuals [3]. Kindness and compassion are among the most valued of human traits and are conducive to well-being [4]–[5]. Studies show that both giving and receiving kindness and compassion have major effects on physiological states and well-being [6]–[10]. As affiliative relationships have a variety of health and well-being benefits and regulate a number of physiological processes throughout life [11], it is important for research to illuminate how affiliative displays are communicated and received.

To be able to orientate towards altruism and compassion requires an ability to attend to it in the verbal and non-verbal presentations of others. A major way we communicate emotions and intentions is via our facial expressions [12]. In social interactions, approval and disapproval are signalled by facial expressions [13]. However, the study of more complex and subtle emotional displays like compassion and kindness is in its infancy, since much emotion research has focused on primary or basic emotions such as anger, fear and happiness [14]–[17], rather than more blended, day-to-day social communications which are utilised in more complex and subtle facial expressions (such as kindness, compassion, shame and contempt). Basic emotions (e.g. anger, disgust, happiness) are said to have evolved to address urgent threats and opportunities related to survival and reproduction [18]. In contrast, self-conscious emotions (e.g. shame, guilt, compassion) are said to have evolved to deal with threats and opportunities related to social interactions and to be involved in regulating social behaviour, cooperation, affiliation and maintaining supportive and helpful social relationships [8], [19]–[20]. Adolphs (2002) suggests that these social emotions are underrepresented in research studies and their role in regulating a wide range of social behaviours has not been fully appreciated. Other researchers have suggested that there are a wide range of positive affect displays beyond the basic display of happiness which are yet to be explored in research [21]–[24]. Some researchers have attempted to generate stimuli which go beyond the basic emotions, such as Dandeneau, Baldwin, Baccus, Sakellaropoulo, and Pruessner’s (2007) stimulus set featuring rejecting and accepting facial stimuli. Haidt and Keltner (1999) also produced sympathetic/compassionate stimuli (these terms were used interchangeably) but only two such photographs were produced in their study.

To date research into the processing of positive facial expressions has used stimulus sets which typically display broad-smiling happy or joyful facial expressions. However, the use of happy faces can be problematic because recent research suggests that the ‘full-smile’ of a happy/joyful face can actually be aversive, and processed as a threat by some individuals [25]–[26]. Schultheiss and colleagues (2005, 2007) suggest that this is because some types of smile - especially broad smiles - communicate social dominance; hence smiles can be aversive. Consistent with this, some researchers suggest that the social dominance communicated in a smile can make others respond with submissiveness and feelings of inferiority [27]–[28]. In addition, although smiles can signal affiliation and social approach, researchers have suggested that for some individuals (e.g. those with social anxiety or high self-criticism), affiliation and social approach can be threatening [29]–[32]. For some individuals, smiles may also be misinterpreted as mocking [29], [33]–[34]. This relates to an issue identified by Ekman (1992) that there are many different types of smile and not all of them communicate positive emotions. Indeed, happy and joyful faces do not necessarily convey kindness or offer feelings of affiliation and safeness. As such, more complex emotional displays such as compassion and kindness are needed, yet there is currently no stimulus set available to researchers containing facial expressions of altruistic/affiliative emotions.

The above further indicates that there may not only be different types of positive emotion displayed on the face, but that individuals may respond to different types of positive emotional display in different ways dependent upon certain predispositions (e.g., anxiety/depression). Some studies have found diminished attention to happy facial expressions in individuals higher in anxiety [35]–[36] or higher in avoidant attachment style [37]. There is also evidence that depressed individuals have difficulties in accurately and quickly recognising happy faces [38]–[39] and frequently judge them as neutral [40] or less positive [41] compared with non-depressed individuals. There is also evidence that individuals with high self-criticism and/or psychopathology have difficulties with processing and receiving kindness and compassion [10], [42]–[44]. Hence it is also important to consider individual differences in the processing of emotional expressions.

Therefore the purpose of the current research was to: i) develop and validate a stimulus set for use in investigating more subtle emotions; and ii) further explore the validity of the stimulus set using well-tested visual-probe methodology to assess its effectiveness in influencing attentional orientation.

## Study 1

The purpose of this first study was to develop a more appropriate stimulus set for use in exploring the processing of affiliative emotions. Specifically, a facial stimulus set was developed depicting three social affects: compassion/kindness, criticism and neutrality.

Definitions of kindness, compassion and friendliness are complex and overlapping. Some authors suggest that in Western societies kindness is commonly used synonymously with the concept of compassion [45]. Although the word compassion can be linked to empathy as its Latin origin “to suffer with” implies, in both Eastern and Western societies, compassion is seen as a much broader multi-faceted concept that includes capacities for the expression of kindness, caring and altruistic concerns. This is essentially the view of researchers whose definition of compassion encompasses a sense of ‘loving-kindness’ and an investment and interest in the nurturance and well-being of another person [6]–[7], [46]. Hence in Study 1 we aimed to create stimuli which convey compassion in terms of the intention of the expresser, and specifically, of there being a desire to present oneself as a kind individual with concern for the well-being of others.

It is important to note that by their very nature the stimuli may be harder to define and label than basic emotion stimuli as they constitute higher-order emotions (i.e. composites of Ekman & Friesen’s (1976) basic six emotions). This said, a stimulus set of subtle and complex emotions has the advantage of being: i) more ecologically valid in everyday situations than basic emotion stimuli; ii) more representative of emotion displays in attachment/affiliative relationship interactions; and iii) more suitable for use with certain populations (e.g. less threatening to those higher in self-criticism, depression and anxiety).

In addition to generating kind and compassionate expressions, we were also interested in generating critical facial expressions rather than angry ones because anger can denote high levels of arousal and potential violent intent [12]. In contrast, critical facial expressions are textured by different social emotions such as contempt and disdain, indicating negative judgements by the expresser. Critical facial expressions are also probably more subtle and common in day-to-day conflicts than aggressive or violent expressions. Moreover, we would argue that contempt and disdainful critical expressions are more common opposites to compassionate and kind ones than expressions of anger or fear.

In previous studies developing facial stimuli, researchers have asked posers/actors to create facial expressions in a variety of ways. These include simply instructing the poser to produce a particular facial expression in a prototypical fashion (e.g., “make a happy face”) [17]; instructing the poser to voluntarily move certain facial muscles in accordance with the expression (e.g., raising the corners of the mouth upward) [15], [47]; asking the poser to evoke the emotion associated with the expression [16] or using facial morphing of the poser’s expressions (e.g., composites of several photographs are produced - see [48]. In regard to the first two methods, although most people have a reasonable ability to voluntarily control their facial expressions, there are some subtle signals (which rely on facial muscles not under our voluntary control) that we can only display when we *feel* the emotion [49] Duchenne de Bologne, 1860). Duchenne de Bologne (1860) found that when participants tried to pose or ‘fake’ a smiling face, they were able to incorporate the muscles around the mouth (zygomaticus major muscles which are under voluntary control) which pull the lips outward and upward. However, they were not able to incorporate the muscles around the eyes (the orbicularis oculi which are not under voluntary control) which push up the cheeks and produce a crease under the eye-lid and ‘crows-feet’ around the eyes. Research has shown that genuine ‘Duchenne’ smiles are distinct from posed smiles [50]–[53]. Distinguishing genuine from fake facial expressions allows individuals to maximally distribute their affiliative efforts towards others displaying genuine affiliative cues, as to direct efforts and resources towards those displaying fake cues would be costly. Hence study 1 combined the use of imagery and emotional memories in generating facial expressions which should result in more genuine and ecologically valid emotional expressions.

To sum, given the evidence that posed smiles are distinguishable to some degree from genuine smiles, study 1 used a carefully designed guided imagery procedure where actors posing the emotions would *feel* the emotions associated with each of the desired expressions.

### Methods

#### Actors and stimulus development

**Figure 1:**
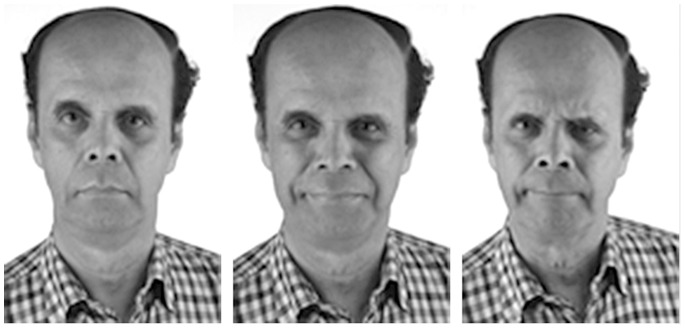
Example of each emotional expression (neutral, compassionate, critical).

A total of 62 actors (757 photographs) from an acting degree course at the University of Northampton participated by posing for the three facial expressions - neutral, compassionate/kind, and critical, in that order. This was following comments obtained at a pilot photography session that emotions of criticism contaminated the other emotion displays if posed first. An example of the expressions posed by one actor is shown in Figure 1. The actor has given written informed consent, as outlined in the PLOS consent form, to publication of their photograph. To enable the actors to stimulate the inner emotions appropriate to the emotional expressions, they were instructed to use imagery and emotional memories of recalling a time when they felt very kind, or critical, towards somebody (inspired by imagery used in Compassion-Focused Therapy; [54]). The use of imagery and emotional memories are frequently used techniques in acting [55] but have not to our knowledge been used together previously in developing facial expression stimuli. In this study, the first author guided the actors through the two minute imagery tasks whilst a professional photographer took repeated photographs in a lighting controlled photography studio. Full imagery instructions are available from the author.

#### Participants in the validation procedure

Psychology students and staff (*N* = 87) from the University of Derby participated in the validation procedure of which 62 participants returned fully completed data. However, two of these participants were identified as extreme outliers (more than two SD’s below the mean score for critical faces). Therefore the final sample consisted of 60 participants (49 females and 11 males), with an age range of 18–60 years (*M* = 32.35, *SD* = 11.60). Men and women did not generally differ in their ratings but men rated critical faces as more ‘neutral’ and women as more ‘other’. Ethical approval was obtained from the University of Derby Department of Psychology. Participants gave written consent to participate. Research commenced in 2007.

#### Materials and Methods

In preliminary analyses, stimuli from nine actors were removed because the panel of researchers felt that they showed little distinction between the three emotions or that their poses were unclear examples of the desired facial expressions. Thus, the final stimulus set of 212 greyscale stimuli put forward for rating in the validation procedure included photographs from 53 actors (212 photographs) comprising: 31 females; 22 males; 35 young actors; 18 mature actors; 49 Caucasian actors; 2 black actors; and 2 Asian actors.

Participants were asked to rate the strength of each emotion type (‘Compassion/warmth’, ‘Neutrality’, ‘Criticism’, ‘Excitement/happiness’, ‘Other Emotion’) present in each photograph on a 0–10 scale (0 = Not present; 1 = Very Mild; 10 = Very Strong). There is some discussion in previous literature [56]–[57] of which method to use to establish how people recognize emotions in photos of facial expressions. Hence our choice of using a detailed quantitative system was adopted given concerns in the literature over free-labelling and forced-choice methodologies [17], [57]–[58].

Participants were seated in a small lecture theatre and the stimuli were presented in a pseudo-random order (so that the same actor did not appear consecutively) via PowerPoint on a display screen. We chose to present photographs in isolation so that other photographs would not provide an emotional context for judgments. The participants gave their rating responses on a paper copy of the ratings manual (copy of rating scale available from corresponding author on request).

To account for any potential biasing effects of individual differences on the participants ratings of the facial expressions, participants were asked to rate to what degree they generally viewed other people as being unfriendly or friendly (1 = Unfriendly 10 = Friendly) and to what degree they were generally critical or kind (1 = Critical 10 = Kind) to themselves on a 1–10 semantic scale. These two dimensions were chosen as they give a broad indication of how participants relate to themselves and others. Bonferroni corrected independent *t* tests showed there were no significant differences in the ratings assigned to facial expressions based on these individual differences.

Means and standard deviations were calculated for the ratings of all 53 actors (212 photographs). Only actors for whom the three expressions were clearly recognised were retained. That is, of the 53 actors, 31 actors (93 photographs, 17 women, 14 men, 21 young, 10 mature, 27 white, 2 black, 2 Asian) had a mean rating of 4 or higher in each of the compassionate, critical and neutral expressions and consisted of the final set of stimuli on which we conducted our analyses.

### Results

The overall mean rating scores for the three expression types across the final 31 actors are presented in Table 1.

**Table 1:** Mean (SD) statistics for the ratings of different types of facial expressions.

Face Type	Emotion Labels				
	Compassion/warmth *Mean (SD)*	Excitement/happiness *Mean (SD)*	Neutrality*Mean (SD)*	Criticism*Mean (SD)*	Other*Mean (SD)*
**Compassionate**	5.82(1.26)	4.37 (1.59)	2.26 (1.94)	0.73 (0.79)	1.17 (1.30)
**Neutral**	1.57 (1.14)	0.85 (1.07)	5.14 (2.03)	2.44 (1.54)	1.93 (1.77)
**Critical**	0.89 (0.70)	0.62 (0.64)	2.07 (1.25)	5.90 (1.42)	3.98 (2.16)

**Note**: Ratings were made on a scale ranging from 0 = not present to 10 = very strong.

Three separate one-way repeated measures ANOVAs were conducted, one for each face type (compassionate, neutral and critical). The repeated-measures factor was Emotion Label with five levels (compassion, neutrality, criticism, happiness/excitement, ‘other’). The dependent variable was the rating score. The ANOVA results indicate that there were significant differences between the mean ratings for emotion label in compassionate expressions [*F* (4,236) = 177.49; *p*≤.001]; neutral expressions [*F* (4,236) = 177.49; *p*≤.001]; and critical faces [*F* (4,236) = 69.92; *p*≤.001]. For each analysis, the Bonferroni corrected post hoc simple contrast tests elucidated that the ratings for the emotion label of the intended emotion significantly differed from the ratings for all other emotion labels (all *p*s ≤.001). In other words, the face types were rated as having the highest degree of their intended emotion and this was significantly different to ratings given for other emotion labels present in the photographs.

#### Retest reliability

To assess retest-reliability, students (*N* = 20) from the original sample were approached four weeks later and asked to rate 50 randomly selected photographs from the stimulus set a second time. Again, participants were asked to rate the strength of each emotion type (‘Compassion/warmth’, ‘Neutrality’, ‘Criticism’, ‘Excitement/happiness’, ‘Other Emotion’) present in each photograph on a 0–10 scale (0 = Not present; 1 = Very Mild; 10 = Very Strong). The correlations between original mean ratings of the intended emotion and retest mean ratings were: *r = *.85 (time 1: *M* = 5.71, *SD* = 1.15; time 2: *M* = 5.65, *SD* = 1.54) for compassionate faces; *r = *.77 (time 1: *M* = 6.73, *SD* = 1.46; time 2: *M* = 6.69, *SD* = 1.54) for critical faces; *r = *.60 (time 1: *M* = 5.16, *SD* = 1.65; time 2: *M* = 5.90, *SD* = 1.87) for neutral faces. It is important to note that in this retest, as in the first testing session, we were not asking individuals to rate whether a face is in a specific category (e.g., compassionate, neutral, critical), but rather to provide an actual score to indicate degree of emotion type present in a facial expression. As far as we are aware this is the first time reliability for facial stimuli has been assessed like this and it is likely that there will be subtle variations in the degree to which individuals rate particular facial expressions.

#### Valence and arousal

Dimensions of valence (i.e. whether a stimulus is perceived as positive or negative) and arousal (i.e. energetic intensity of stimuli) are thought to underlie approach and avoidance behaviours and play a critical role in directing attention [59]. Hence independent judges (*N* = 9) provided ratings of valence (1 = negative to 10 positive) and arousal (1 = low arousal to 10 = high arousal) for the final stimulus set. A Kruskal Wallis analysis of the ratings revealed that there were significant differences in the ratings of valence (*H* (2) = 22.33, *p*≤.01) and arousal (*H* (2) = 15.81, *p*≤.01) for compassionate, critical and neutral expressions. These significant differences were supported between all three face types (compassionate, critical and neutral) by post hoc Mann-Whitney U tests (all *p*s ≤.05). As expected, compassionate expressions were rated as having positive valence (*M* = 7.09, *SD* = .34) and moderate arousal (*M* = 4.69, *SD* = .76); critical expressions were rated as having negative valence (*M* = 2.99, *SD* = .82) and higher arousal (*M* = 6.18, *SD* = 1.17); and neutral expressions were rated as having neutral valence (*M* = 4.47, *SD* = .35) and low arousal (*M* = 3.54, *SD* = 1.14).

### Discussion

This study developed a new facial stimulus set featuring facial expressions of kindness-compassion, criticism and neutrality as no such stimulus set currently exists in the literature. High-resolution greyscale photographs of faces were created using a carefully designed *imagery and emotional memory procedure* with a group of actors. This procedure aimed to *create* emotions in actors rather than simply asking them to pose emotions. The results of this study indicate that the facial stimuli were accurately and reliably identified. Thus we have developed a valid stimulus set (based on 31 actors) comprising highly recognisable facial expressions of compassion, criticism and neutrality as rated by an untrained sample. It is important to note that for this new stimulus set, all facial expressions received the highest mean ratings for the intended facial expression and that these ratings were *significantly* higher compared to the ratings for other emotion labels present in each photograph. In terms of overall mean scores for each emotion type, critical facial expressions received the highest ratings for the intended facial expression, followed by compassionate expressions and finally neutral expressions. Valence and arousal ratings indicated that compassionate, critical and neutral faces were distinct, with compassionate faces rated as high in positive valence and moderate in arousal; critical faces were high in negative valence and arousal; and neutral faces were moderate in valence, but lower in arousal. In addition, the highest retest reliability was found for compassionate expressions and the lowest retest reliability was found for neutral expressions. The lower retest reliability for neutral faces is not surprising because of the previously reported ambiguity of neutral facial expressions [60].

To sum, the overall findings are that expressions which were created to signal certain types of emotion (e.g. compassion, criticism) can be reliably detected by independent raters. Importantly, for our study, efforts to create images of compassionate/kind faces were successful and they were clearly distinguished from images of critical and neutral faces. This stimulus set (known as the ‘McEwan Faces’) with mean ratings included is available on request from the corresponding author KM at the Centre for Psychological Research at the University of Derby.

## Study 2

To further validate the McEwan Faces stimulus set and demonstrate its effectiveness, in study 2 we used the stimulus set in a visual probe task to investigate the processing of compassionate/kind and critical faces in relation to self-criticism and mood. We chose to do this by utilising the visual probe task. This is a well known cognitive paradigm used to investigate attentional biases in emotion processing [29], [61]–[62].

Computerised visual probe tasks, where participants’ reaction times to probes (usually dots) replacing an angry or neutral face are measured, have frequently been employed to explore selective attention (also known as attentional bias/orientation) to threatening stimuli such as angry faces [61]. Probes are responded to faster when they appear in an attended location, thus giving an idea of where attention is allocated.

There is now a wealth of research focusing on the processing of threatening emotional displays. The majority of research shows evidence of prioritised processing and biased attention toward threatening facial expressions, compared with neutral or positive expressions [29], [63]–[65]. Yet, the processing of positive emotional displays especially higher-order, complex, and blended displays such as compassion and kindness has received limited consideration [6], [23], [24]. This may be because of the lack of valid stimuli.

In addition, in the study of complex emotional stimuli, such as compassion, it may be informative as well as important to consider individual differences. Researchers are increasingly finding that some individuals have difficulties in detecting and responding to compassion from others. For example, Rockliff et al. (2008) found that imagining somebody being kind to oneself increased heart rate variability (indicative of physiological soothing) and reduced cortisol (a stress hormone) in people with lower self-criticism but reduced heart rate variability and produced no change in cortisol in individuals with higher self-criticism. Similarly, in an fMRI study, Longe et al. (2010) found that when asked to be self-reassuring in a threatening scenario, individuals higher in self-criticism showed activation within brain areas associated with threat (e.g., amygdala). Clinical researchers have also found that depressed individuals can struggle with generating feelings of self-compassion, or being open and sensitive to the compassion of others (including the clinician), [44]. This tendency for those with certain traits such as higher self-criticism to struggle to process compassion may translate into an emotion-congruent effect [66] on attentional processes i.e. those higher in self-criticism may attend less to compassionate faces and attend more to critical faces or in other words, demonstrate a bias away from compassionate faces and a bias towards critical faces.

Therefore in Study 2 we used visual probe methodology to explore orientation towards compassionate as well as critical facial expressions. We hypothesised that because compassion is an emotional expression which characterises supportive and loving relationships, it should elicit a sense of safeness and security for *most* recipients and thus avoid the issues associated with happy faces (e.g. being processed as a threat, e.g., [25]). In other words low-self critics (secure individuals) should demonstrate a bias towards such compassionate stimuli. However, based upon the above individual difference research, we further hypothesised that individuals with high self-criticism and/or low mood may respond to compassion as though it were a threat and thus display no bias towards, or a bias away from, such stimuli.

### Methods

#### Participants

Participants were 70 psychology undergraduates studying at the University of Leicester. Data from two participants was later excluded because of extreme outlier reaction times (i.e., three SD’s above or below the group mean), leaving 68 participants. There were 54 females and 14 males, their ages ranged from 18–45 years (*M = *20.53, *SD = *20.53). Ethical approval was obtained from the University of Leicester Department of Psychology. Participants gave written consent to participate. Research commenced in 2009.

#### Methods and procedures

Participants completed a computerised visual probe task which used the McEwan Faces stimulus set. The facial stimuli were presented in greyscale and had a resolution of 72 dpi, they measured 45×70 mm on the computer screen and had a distance of 115 mm between their centres. Participants were then asked to complete a series of questionnaires which included the Forms of self-criticism/reassurance scale [67] and the Depression, anxiety and stress scale [68].

The VPT involved participants responding (by pressing keys on a button-box) to a visual probe (a pair of dots) to indicate which probe (i.e. **:** or **.** ) replaced one of a pair of photographic facial stimuli. Participants were given 16 practice trials followed by 64 experimental trials (16 compassionate-neutral pairs and 16 critical-neutral pairs presented twice in both left- and right-visual fields). Each trial started with a fixation point presented for 500 ms at the centre of the screen. This was then replaced with a pair of facial stimuli, and finally by the probes replacing one of the pair of stimuli. The inter-trial interval varied randomly between 500 ms and 1250 ms as in previous visual probe methodologies [69]. The VPT was programmed using E-Prime software and was presented on a PC computer with a 15-inch monitor. Both the time it took participants to press a key on the button-box and accuracy of response were recorded. When the probe replaces the emotional face, this is known as a valid trial and a fast reaction time to a valid trial indicates engagement with, or enhanced attention to, this face. When the probe replaces the neutral face, this is known as an invalid trial and a fast reaction time to an invalid trial indicates disengagement with, or diminished attention to, the emotional face in order to respond to the neutral face. For more detail on the VPT task specifics see [69].

After completing the computerised visual probe task participants were asked to complete a series of questionnaires which included the Forms of self-criticism/reassurance scale [67] and the Depression, anxiety and stress scale [68]. To assess the effect of these individual differences (i.e. self-criticism and current mood (anxiety)) on the processing of compassionate and critical facial expressions, median-split methodology was used [64] to allocate participants to a low or high self-criticism group (median cut point score of 16), and a low or high anxiety group (median cut point score of 16) for analysis. Level of self-criticism and anxiety (low vs high) were entered as between-subjects variables in the following analyses.

The VPT data were screened for participant errors in responding, and reaction time (RT) outliers. Data from trials where participants had made errors in responding were discarded (2.61% of trials), as were data from trials where RTs were: i) less than 200ms; and ii) greater than two standard deviations above each participant’s total mean RT (3.54% of trials).

Attentional bias scores were then calculated for the compassionate/kind faces trials and the critical faces trials, employing the same procedure as MacLeod et al. (1986). The normality of distributions for RTs and questionnaire measures were good (skewness = .12 to 1.25 & kurtosis = −.17 to 1.00). The two attentional bias scores were entered as dependent variables in the following analyses.

### Results

Mean RTs when probes replaced compassionate faces, critical faces and neutral faces were 600.27 ms; 598.35 ms and 602.76 ms respectively. The mean attentional bias scores for critical faces were *M = *1.78, (*SD = *25.23) and for compassionate faces *M = *0.35, (*SD = *22.85).

Two univariate ANOVA’s were conducted with level of Self-Criticism (low vs. high) as the independent variable and the Bias scores for face type (critical or compassionate) as the dependent variables. Table 2 presents means and standard deviations per condition. The univariate analyses revealed that for the critical face bias score, no significant effect of level of self-criticism was found (*p*≥.1). However, for the compassionate face bias score a significant effect of level of self-criticism was observed [*F* (1, 66) = 6.37, *p*≤.05, η_p_^2^ = .088]. Mean bias scores demonstrated that higher and lower scorers on self-criticism differed significantly in their attentional bias towards compassionate facial expressions. That is, the high self-criticism group appeared to show a negative bias (i.e. diminished attention; or attention away from) for compassionate facial expressions, whereas the low self-criticism group appeared to show a positive bias (i.e. enhanced attention) to compassionate faces.

**Table 2:** Attentional bias score means and standard deviations per condition.

	Bias scores	
	Critical*Mean (SD)*	Compassionate*Mean (SD)*
**Low self-criticism**	6.83 (25.21)	7.70 (18.11)
**High self-criticism**	−2.44 (24.80)	−5.81 (24.76)
**Low anxiety**	4.35 (23.70)	3.33 (19.26)
**High anxiety**	−.24 (26.51)	−2.01 (25.34)

To further investigate this, difference from zero was assessed for compassionate faces [69]. That is, one-sample t-tests were conducted for both higher and lower self-criticism groups comparing their mean attentional bias scores to “0”, the theoretical *non-*bias score reference point. An attentional bias score of “0” represents equal reaction times to invalid and valid trials, thereby indicating *no* bias toward or away from facial expressions. Analyses showed that low self-critics differed significantly from 0 (*t* (30) = 2.37; *p* = .025), whereas high self-critics did not (*p*≥.1). Thus, lower self-critics showed enhanced attention toward compassionate faces whereas those higher in self-criticism did not.

Two univariate ANOVA’s were conducted with level of Anxiety (low vs. high) as the independent variable and the Bias scores for face type (critical or compassionate) as the dependent variables. There were no significant effects of level of anxiety (*p*≥.1).

#### Additional tertile analyses

Although using median-splits of individual difference scores is a common method of analysis in VPT studies [29], [62], [64], [70], we are aware that some researchers debate their use [71]. Median-splits were used in the current analyses to replicate the analyses of previous studies and allow comparison with previous findings. However, we also conducted tertile analyses where questionnaire scores are divided into low (0–15), medium (16–23) & high (24–34) to see whether the findings can be replicated. These analyses replicated the main effect of self-criticism [*F* (4, 130) = 3.13, *p*≤.05, η_p_^2^ = .088] and showed that higher scores in self-criticism are associated with negative biases (i.e. diminished attention) toward compassionate faces [*F* (2, 65) = 3.15, *p*≤.05, η_p_^2^ = .088]. One-sample t-test (comparing bias score to zero) findings were also replicated.

### Discussion

In Study 2 the new McEwan facial stimulus set developed and validated in study 1 was used in the well-established visual probe task to assess processing of compassionate and critical faces. It was found that self-criticism significantly affected how facial expressions are processed; namely those lower in self-criticism showed enhanced attention to compassionate faces whilst in contrast, those higher in self-criticism showed no bias (or diminished attention) to compassionate faces. This finding is consistent with the emotion-congruency perspective of attentional bias whereby state or trait characteristics (such as self-criticism or anxiety) predispose individuals to focus their attention on information congruent with that state or trait [66]. For example, many studies of attentional biases show congruency effects in terms of anxious individuals attending to threatening information [61]–[62], [65]; depressed individuals attending to depression or failure-related information [72]; individuals with obsessive-compulsive disorder attending to contamination information [73]; and optimistic individuals attending to positive information [74]. The tendency for those lower in self-criticism to attend towards the compassionate facial expressions fits with this, as does the finding of diminished attention towards compassionate facial expressions in those higher in self-criticism.

Indeed, whilst this latter finding needs further exploration, a possible explanation for it could be that those higher in self-criticism may experience a conflict between desiring social engagement/affiliation-seeking, as well as a fear of disappointment and rejection [75]. In other words, the more the affiliative system is active then so too is the threat system [10], [43], [54]. This diminished attention is consistent with previous research which shows that those higher in self-criticism have difficulties receiving compassionate cues, even imagined ones [10], [43].

Finally, we note that there were no effects for the individual difference of anxiety. This could be due to the low levels of anxiety in this healthy student population (which were within the ‘normal-mild’ range as defined by clinical cut-offs; [68]). Certainly, it is common in VPT studies that attentional biases are only revealed in a healthy population where stress has been induced prior to testing.

## General Discussion

The aims of the present research were twofold: i) to develop and validate a facial stimulus set of subtle higher emotions, as to date no stimulus set displaying more subtle emotions exists; and ii) to investigate attentional orientation to facial expressions of subtle emotions in relation to self-criticism and mood. To this end, study 1 developed and validated the first stimulus set (to our knowledge) to include higher order, complex positive affect displays such as compassion. In the development of this stimulus set great care was taken to ensure that expressions were ecologically valid, by not only using actors in the development process but also by incorporating methods of emotional memory and imagery to generate the expressions. The new stimulus set – the McEwan Faces - was then used in study 2 to investigate attentional processing. In this study, consistent with hypotheses, it was found that self-criticism significantly affected how facial expressions are processed. Lower self-criticism was associated with a positive bias (i.e. enhanced attention) to compassionate faces whilst higher self-criticism was associated with diminished attention or no bias to compassionate faces. This latter finding fits well with previous research that has demonstrated high self-critics generally struggle to engage with compassion [10], [43], [54].

Of importance, it is notable that these results were found in this healthy population with no prior mood induction techniques [35]–[36]. Typically, attentional biases are only found in clinical samples where some form of psychopathology or social anxiety is currently being experienced [29], [61]–[62], [65], [72] unless mood/stress induction techniques are used [36]. In addition, the new stimulus set consists of more complex and subtle emotional expressions compared with previous research which has utilised basic and prototypical emotional expressions (12, 15–17].

That a significant attentional bias found in this study was for positive (compassionate/kind) faces is especially important, as previous studies typically reveal attentional biases for threatening facial expressions such as anger only [61]–[62]. Few studies find biases for positive (happy/joyful) facial expressions [29], [70]. Critically, this may reflect the importance of distinguishing compassionate and kind expressions from other positive emotions or the fact that certain positive facial expressions are processed differently from others and may even be processed as threatening. However, that our critical faces did not produce significant effects may reflect their subtlety and that they may not stimulate basic fight or flight responses but rather more complex negative social emotions. In other words, unlike angry (or fearful faces), critical faces may not be perceived as an immediate, direct threat and therefore would not activate hypothesised threat superiority mechanisms [76].

Although the findings of our Study 1 and the pattern of results of Study 2 strongly suggest that the newly created stimulus set validly depicts the intended complex emotions, future research needs to further explore the relation of verbal emotion labels to facial expressions. For example, what makes these expressions distinct from other expressions, maybe in terms of ‘action units’, could be explored by use of the Facial Action Coding System (FACS-[77]).

One key advantage of the new stimulus set is that it is the first (to our knowledge) to include higher order, complex affect displays such as compassion and criticism. Thus, this stimulus set can be used in emotion processing research to further investigate processing of affiliative relationships as conveyed through non-verbal displays such as compassionate faces. This is important because happy faces are not the same as kind-compassionate faces and researchers [27]–[29], [25]–[26] have found that some individuals can find ‘happy faces’ threatening. By using this new facial expression stimulus set, future research can explore responses to complex positive facial stimuli that are less likely to be threatening to such individuals.

To conclude, it is evident that our stimulus set developed by using methods of emotional memory and imagery produced interpretable findings in a visual probe task. This new stimulus set can therefore be used in emotion processing research to further investigate processing of complex emotions and affiliative relationships across a variety of different cognitive testing paradigms.
